# Administration of heat killed *Fructobacillus fructosus* OS-1010 attenuates metabolic disease induced by high fat diet in mice

**DOI:** 10.1038/s41598-025-14312-5

**Published:** 2025-08-09

**Authors:** Yoshiyuki Nakano, Ryosuke Nakamura, Hina Tanaka, Yuji Tokimoto, Yuna Masuda, Noriaki Emoto, Kouji Nishikawa, Hideaki Idogaki

**Affiliations:** 1 Innovation Center, Osaka Soda Co., Ltd, 9 Ohtakasu-cho, Amagasaki, Hyogo 660-0842 Japan; 2 Laboratory of Clinical Pharmaceutical Science, Kobe Pharmaceutical University, 4-19-1 Motoyamakitamachi, Higashinada, Kobe, Hyogo 658-8558 Japan

**Keywords:** Lactic acid bacteria, Heat-killed *F. fructosus* OS-1010, Obesity, Non-alcoholic fatty liver disease, Skeletal muscle dysfunction, Mitochondria, Nutrition, Obesity

## Abstract

Heat-killed *Fructobacillus fructosus* OS-1010 reportedly enhance the number and membrane potential of mitochondria in muscle cell C2C12 in vitro. However, there are no reports on the effects of this strain on mitochondria or the resulting effects on the body in animal models. In this study, we investigated the effects of heat-killed *F. fructosus* OS-1010 on obesity and other metabolic abnormalities and muscle weakness in mice with high-fat diet (HFD)-induced sarcopenic obesity. C57BL/6 mice were fed HFD supplemented with heat-killed *F. fructosus* OS-1010 for 13 weeks. The HFD-induced body weight gain was significantly reduced. Additionally, there was a significant decrease in alanine aminotransferase levels, improvement in serum lipid profiles, and a reduction in non-alcoholic fatty liver disease (NAFLD) progression. Skeletal muscle weakness was also mitigated, with changes in gene expression in the quadriceps indicating suppression of intramuscular fat accumulation, and enhancement of mitochondrial density. The findings of this study suggest that heat-killed *F. fructosus* OS-1010 functions as an anti-obesity postbiotic, potentially benefiting both NAFLD and muscle weakness associated with obesity.

## Introduction

Lactic acid bacteria (LAB) have attracted considerable attention owing to their capacity to enhance well-being, particularly in gastrointestinal health. The health benefits of LAB are primarily attributed to their consumption as probiotics, which are live microorganisms^1^. However, growing interest in postbiotics/paraprobiotics, that is, heat-killed or tyndallized probiotics, has emerged due to concerns regarding the safety of consuming live microorganisms^2,3^. The beneficial effects of heat-killed LAB on metabolic diseases have been the focus of substantial research, with studies demonstrating that heat-killed LAB can reduce body weight gain and lipid metabolism dysfunction in animal models^4–7^. Additionally, heat-killed LAB exert beneficial effects on skeletal muscle health^8^. However, despite this social need, research on heat-killed LAB is limited compared to that on live LAB, and several aspects of their beneficial effects and mechanism of action remain unclear or poorly understood.

*Fructobacillus* is a genus of fructophilic LAB (FLAB) found in fructose-rich environments^9^, and has been isolated from traditional Mexican beverages, such as tarbena, fresh honey, and honey powder^10–12^. Despite the prevalence of FLAB in food sources commonly consumed by humans, which suggests that it may share similar safety characteristics with general probiotic LAB, research on health-promoting effects is insufficient. Under such circumstances, a recent study using an in vitro cell model system revealed a noteworthy phenomenon in which heat-killed *Fructobacillus fructosus* OS-1010, a species of FLAB, induced the mitochondrial biogenesis in C2C12 muscle cells via exosomes derived from *F. fructosus* OS-1010-stimulated Caco-2 intestinal epithelial cells^13^. This study proposed the mechanism involving exosome-mediated promotion of mitochondrial biogenesis, a novel concept that entirely differs from other heat-killed LAB mechanisms of action that have been proposed previously. Mitochondria, which are crucial for energy production in cells, are involved in muscle dysfunction (sarcopenia) associated with obesity and ageing^14^. In light of these findings, it is hypothesized that heat-killed *F. fructosus* OS-1010 affects mitochondrial content in distant tissues via exosome release from the intestinal tract into the bloodstream, potentially offering secondary health benefits, including anti-obesity and improved skeletal muscle function. However, the potential of heat-killed *F. fructosus* OS-1010 to influence the mitochondrial state in distant tissues from intestine, such as skeletal muscle, within the complex biological system of animals, remains to be elucidated. Moreover, the capacity to exert beneficial effects on skeletal muscle function or other metabolic outcomes, such as body weight, lipid metabolism, and liver function, is not yet demonstrated. Therefore, an in vivo study using animal models has been a crucial examination subject in evaluating the potential and efficacy of heat-killed *F. fructosus* OS-1010 as functional postbiotics. In this study, we exploratively investigated the effects of heat-killed *F. fructosus* OS-1010 on metabolic diseases such as obesity, dyslipidemia, and non-alcoholic fatty liver disease (NAFLD) induced by a high-fat diet (HFD), as well as muscle weakness in a male mouse model of HFD-induced sarcopenic obesity. This is the first report to examine the effects of heat-killed *F. fructosus* OS-1010 on metabolism in an animal model, providing preliminary evidence of its potential to alleviate metabolic disease.

## Results

### Heat-killed *F. fructosus* OS-1010 administration reduced body weight gain induced by HFD

**Fig. 1:**
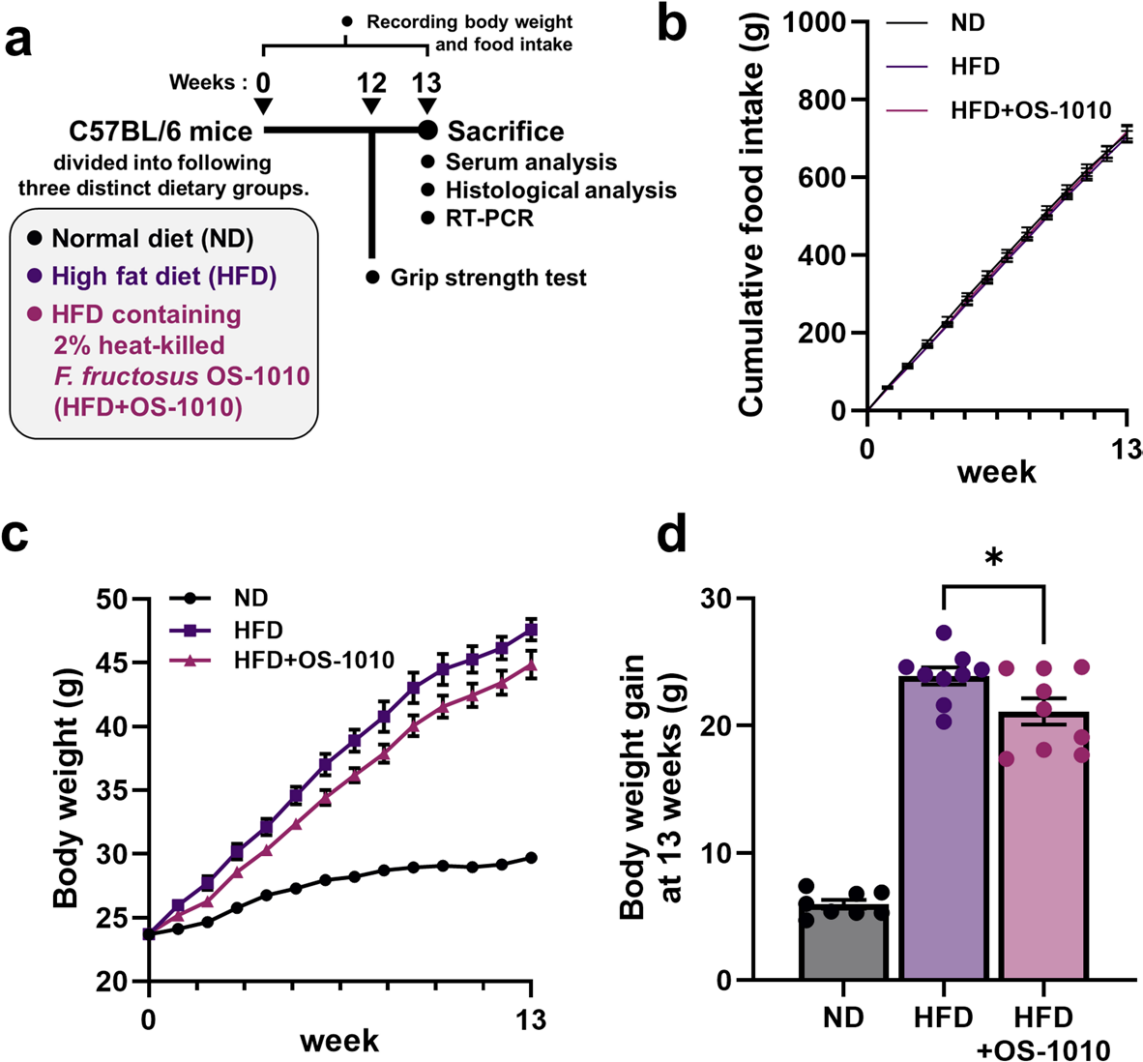
Effects of heat-killed *F. fructosus* OS-1010 on obesity in HFD-fed mice. (**a**) Experimental flow diagram. (**b**) Cumulative food intake over a 13-week feeding period. (**c**) Changes in body weight (*n* = 8–9 animals/group). (**d**) Body weight gain at 13 weeks. All data were presented as mean ± s.e.m. (*n* = 8–9 animals/group). The statistical significance between the HFD group and the HFD + OS-1010 group was calculated using the Mann-Whitney *U* test (*P < 0.05). HFD, high-fat diet; ND, normal diet.

The mice were divided into three groups with distinct diets (normal diet [ND], HFD, HFD + OS-1010; *n* = 8–9/group) and fed until sacrifice (Fig. 1a). No significant difference in food consumption was observed between the groups over the 13-week period (Fig. 1b). HFD + OS-1010 feed is identical in composition to standard HFD feed in terms of calories and nutritional components (including carbohydrate, protein, and fat), except that it contains heat-killed *F. fructosus* OS-1010. The body weight changes in each group showed a different pattern (Fig. 1c). At 13 weeks, the HFD group showed typical body weight gain, increasing by 23.9 g, approximately four times the weight observed in the ND group (5.9 g) (Fig. 1d). Body weight gain is the main parameter for assessing the outcome of obesity development. In contrast, the HFD + OS-1010 group showed a weight gain of 21.1 g, significantly less than that of the HFD group (*P* = 0.0366). These results suggest that administration of heat-killed *F. fructosus* OS-1010 significantly reduced HFD-induced weight gain.

### Improvement of lipid profile in blood

**Fig. 2:**
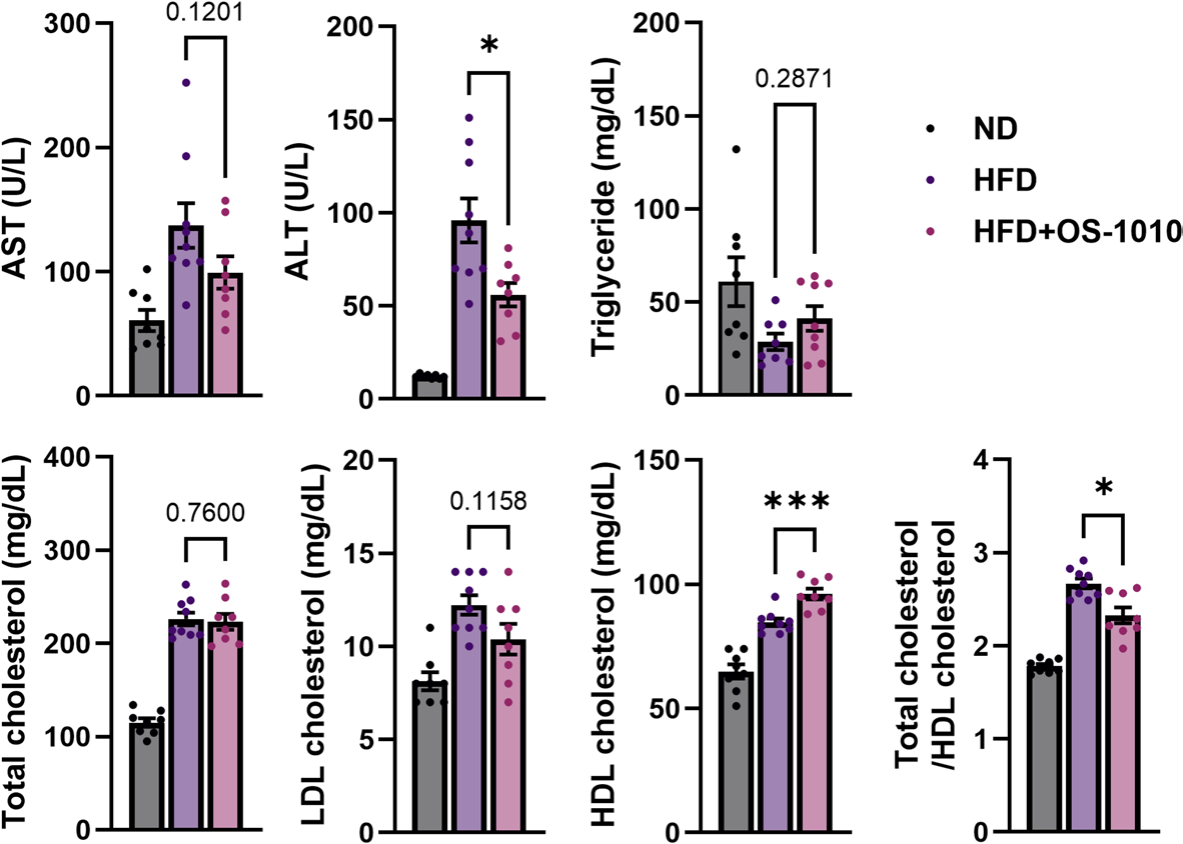
Serum biochemical parameters. All data were presented as mean ± s.e.m. (*n* = 8–9 animals/group). The statistical significance between the HFD group and the HFD + OS-1010 group was calculated using the Mann-Whitney U test (*P < 0.05, ***P < 0.001). ALT, alanine aminotransferase; AST, aspartate aminotransferase; HDL, high-density lipoprotein; HFD, high-fat diet; LDL, low-density lipoprotein; ND, normal diet.

Next, biochemical indicators associated with liver function and blood lipid levels were examined. The effects of the HFD on each indicator were apparent, and in the HFD and HFD + OS-1010 groups, aspartate aminotransferase (AST) and alanine aminotransferase (ALT) increased (Fig. 2), indicating hepatic injury. While these values increased in the HFD + OS-1010 group compared with those of the ND group, the level of ALT in HFD + OS-1010 group was considerably reduced when compared to the value observed in the HFD group (*P* = 0.0139), suggesting a mitigating effect of HFD on hepatic injury.

Triglyceride levels showed a slight decline in response to the HFD, but this shift was not statistically significant, aligning with previous studies in C57BL/6 mice^15^. No significant differences in triglyceride levels were observed between the HFD and HFD + OS-1010 groups. Total cholesterol and low-density lipoprotein cholesterol (LDL-C) levels were elevated in both HFD and HFD + OS-1010 groups compared with those in the ND group, though the difference between the two groups was not significant. Notably, high-density lipoprotein cholesterol (HDL-C) levels increased in response to HFD, with significantly higher levels in the HFD + OS-1010 group compared with that in the HFD group (*P* = 0.0005). Additionally, the ratio of total cholesterol to HDL-C was significantly reduced compared with that of the HFD group (*P* = 0.0206). This ratio has been proposed as an effective index of obesity and hyperlipidemia^16,17^. These results suggest that heat-killed *F. fructosus* OS-1010 improves the lipid profile and significantly mitigates obesity.

### Potential for controlling the progression of non-alcoholic fatty liver disease (NAFLD)

**Fig. 3:**
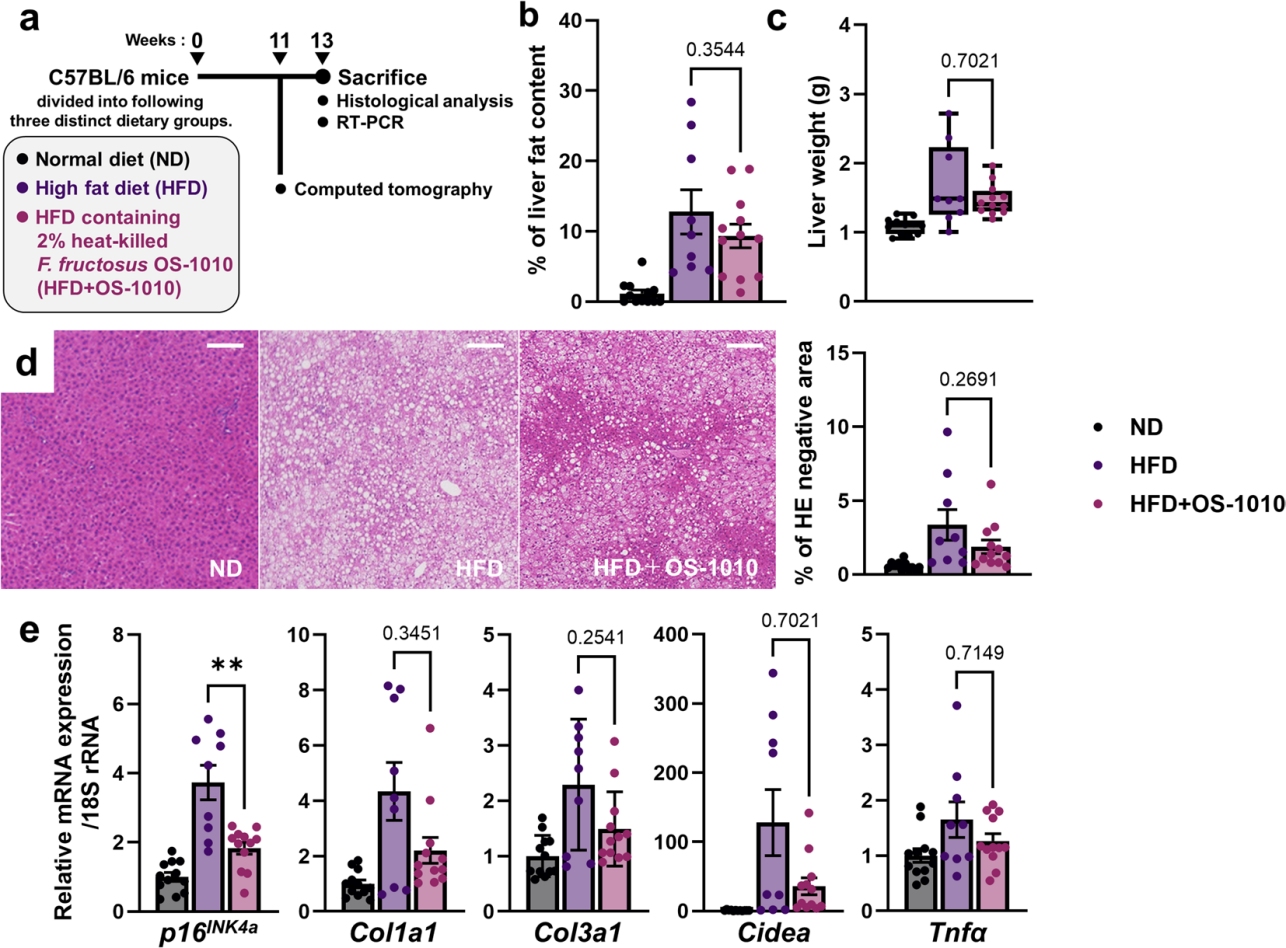
Effects of heat-killed *F. fructosus* OS-1010 on liver (**a**) Flow diagram focusing on experiments with liver. (**b**) CT-estimated liver fat content at 11 weeks. Data were presented as mean ± s.e.m. (*n* = 9–12 animals/group). (**c**) Liver weights are shown as box and whiskers plots (*n* = 9–12 animals/group). (**d**) Representative HE histology sections of liver tissue. Scale bar, 100 μm. (**e**) mRNA expression of genes associated with NAFLD. Data were presented as mean ± s.e.m. (*n* = 9–12 animals/group). The statistical significance between the HFD group and the HFD + OS-1010 group was calculated using the Mann-Whitney *U* test (**P < 0.01). HE, hematoxylin and eosin; HFD, high-fat diet; ND, normal diet.

Following the serological analysis, we performed histological and biochemical evaluations of the liver (Fig. 3a). After 11 weeks of feeding, the degree of fat accumulation in the liver was evaluated using computed tomography (CT), showing progression in both the HFD and HFD + OS-1010 groups (Fig. 3b). No statistically significant difference in fat accumulation was detected between the HFD group and the HFD + OS-1010 group. These results were consistent with the liver tissue weight measurements and oil droplet (hematoxylin and eosin [HE]-negative) area analysis within the tissue (Fig. 3c,d).

Subsequently, we examined the expression of NAFLD-associated genes (Fig. 3e). The expression of cyclin-dependent kinase inhibitor 2 A (*p16*^*INK4a*^), which increases during NAFLD progression^18^ was elevated in both the HFD and HFD + OS-1010 groups. However, this increase was significantly reduced in the HFD + OS-1010 group (*P* = 0.0056). Fibrotic markers, *Col1a1* and *Col3a1*^19,20^, were increased in both groups. Cell death-inducing DFFA-like effector A (*Cidea*), a lipogenic gene involved in NAFLD progression^21^ was notably upregulated in some mice in the HFD group. In addition, the inflammatory marker, *Tnfα*^22^ was upregulated in both the HFD and HFD + OS-1010 groups. Although no significant differences were detected between the HFD group and the HFD + OS-1010 group in the four genes (*Col1a1*, *Col3a1*, *Cidea*, and *Tnfα*), significant *p16*^INK4^ downregulation and improvement in serum ALT level suggest that administration of *F. fructosus* OS-1010 has a potential contribution to the mitigation of NAFLD progression.

### Reduced muscle weakness induced by obesity, but no structural change of muscle

**Fig. 4:**
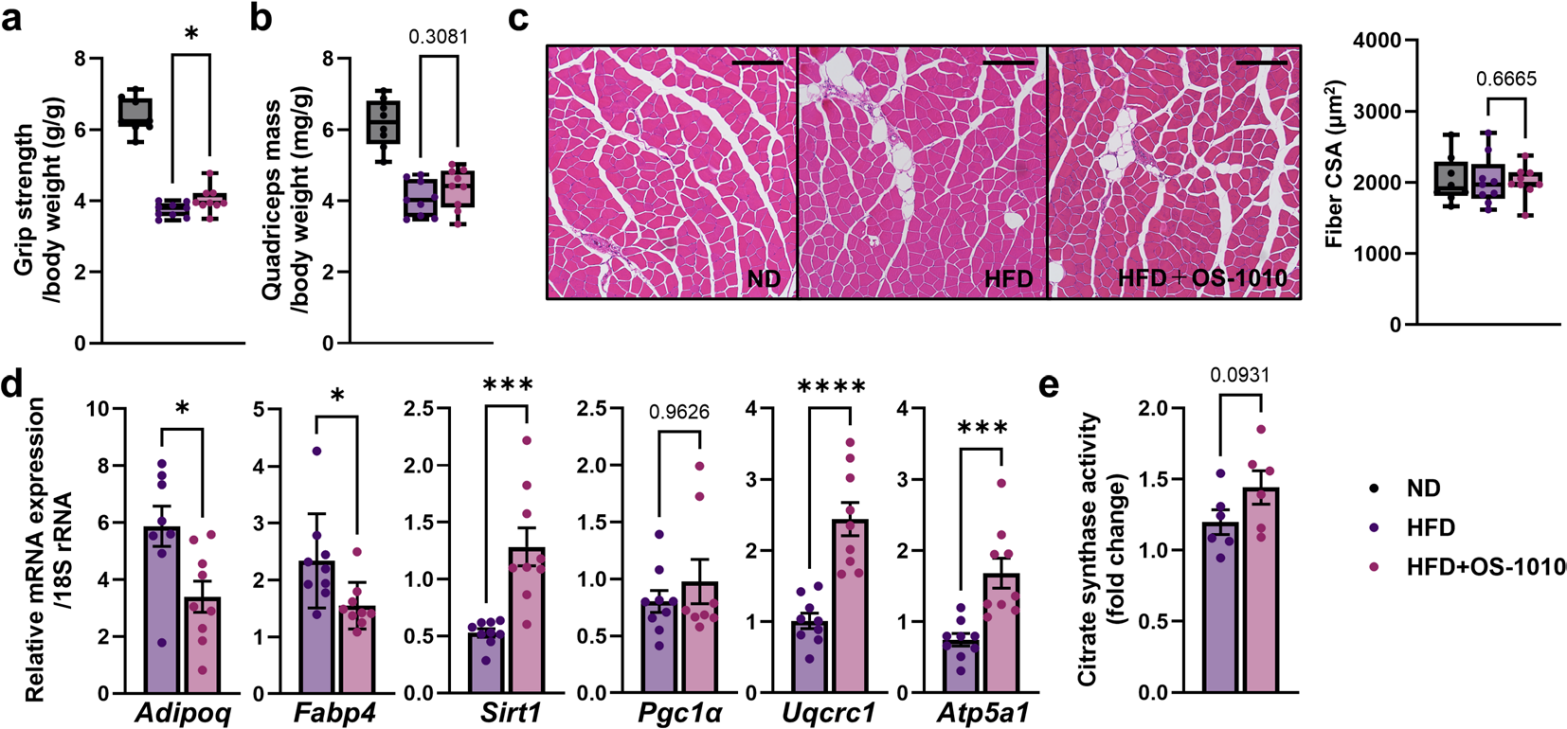
Effects of heat-killed *F. fructosus* OS-1010 on quadriceps. (**a**) Grip strength per body weight are shown as box and whiskers plots (*n* = 8–9 animals/group). (**b**) Wet total quadriceps mass per body weight are shown as box and whiskers plots (*n* = 8–9 animals/group). (**c**) Representative HE histology sections of rectus femoris. Scale bar, 200 μm. Fiber cross-sectional area (CSA) is presented as box and whiskers plots (*n* = 8–9 animals/group). (**d**) mRNA expression of genes associated with intramuscular fat and mitochondria. Data were presented as mean ± s.e.m. (*n* = 8–9 animals/group), and expressed relative to ND group. (**e**)Activity of the citrate synthase. Data were presented as mean ± s.e.m. (*n* = 6 animals/group), and expressed relative to ND group. The statistical significance between the HFD group and the HFD + OS-1010 group was calculated using the Mann-Whitney *U* test (*P < 0.05, ***P < 0.001, ****P < 0.0001). HFD, high-fat diet; ND, normal diet.

Obese mice are known to exhibit muscle dysfunction (sarcopenia), often used as a model for age-related sarcopenia^23^. Grip strength, a primary parameter of skeletal muscle performance, decreased in both the HFD and HFD + OS-1010 groups (Fig. 4a). However, compared with the HFD group, this decrease was significantly mitigated in the HFD + OS-1010 group (*P* = 0.0472).

Given the established relationship between muscle strength and mass, we analyzed the histology of the quadriceps, which is a typical mixed-fiber muscle and metabolically very similar to human vastus lateralis^24^. The HFD led to reduced muscle weight in the quadriceps (Fig. 4b). With regard to the cross-sectional area of the muscle fibers, the HFD had no effect on the rectus femoris (Fig. 4c). There were no significant differences in muscle weight or muscle fiber thickness between the two groups. This suggests that the reduced the decline in grip strength observed in the HFD + OS-1010 group was not due to the maintenance of muscle structure.

### Downregulation of *Adipoq*, and *Fabp4* provides insight into the maintenance of muscle strength

Next, we analyzed the expression of gene related to adipocytes in muscle. Intermuscular adipose tissue (IMAT) accumulation within skeletal muscles negatively affects muscle strength^25–27^. Therefore, we analyzed the expression of *Adipoq* and fatty acid-binding protein 4 (*Fabp4*), which are markers of mature adipocytes in skeletal muscle^28,29^. The expression of these genes was significantly suppressed in the HFD + OS-1010 group (*Adipoq*, *P* = 0.0154; *Fabp4*, *P* = 0.0142, Fig. 4d). These results provide insights suggesting that the accumulation of IMAT was suppressed by the administration of *F. fructosus* OS-1010.

### Upregulation of *Sirt1*, a sirtuin gene involved in the regulation of mitochondrial function and biogenesis

Finally, we analyzed the expression of genes related to mitochondrial biosynthesis, which plays a key role in skeletal muscle function. In the quadriceps, the expression of *Sirt1* was significantly increased (*P* = 0.0003, Fig. 4d). However, we observed no significant change in the mRNA expression of the peroxisome proliferator activated receptor γ co-activator 1α (*Pgc1α*), a transcriptional regulator of mitochondria biogenesis, which is located downstream of the pathway mediated by SIRT1^30–32^. In addition, the expression of *Uqcrc1*, a complex III subunit gene, and *Atp5a1*, a subunit gene of ATP synthase, which are key components of the oxidative phosphorylation (OXPHOS) pathway responsible for ATP production in the mitochondria, was significantly increased (*Uqcrc1*, *P* < 0.0001; *Atp5a1*, *P* = 0.0002). The enhanced transcription of these mitochondrial proteins suggests an increase in the number of mitochondria. Additionally, citrate synthase (CS) activity, an indicator of mitochondrial density, showed a trend towards increased activity in the HFD + OS-1010 group compared to the HFD group (*P* = 0.093, Fig. 4e). These results suggest that the administration of *F. fructosus* OS-1010 has a tendency to improve mitochondrial density in skeletal muscle.

## Discussion

Our findings demonstrated that administration of heat-killed *F. fructosus* OS-1010 has an anti-obesity effect, evidenced by improvements in the blood lipid profile, and reducing body weight gain induced by HFD. There were no differences in food intake between the HFD group and the HFD + OS-1010 group, and since the calories and nutrients in the feed were the same, this anti-obesity effect was suggested to be due to the heat-killed *F. fructosus* OS-1010 administration rather than differences in calorie or nutrient intake. Note that this study used male mice, and it cannot be ruled out that the results may differ due to sex differences.

A significant decrease in ALT levels in the blood and a downregulation in hepatic senescence marker gene *p16*^*INK4a*^, which drives the phenotype of NAFLD^33^ suggest the possibility of suppressing the progression of NAFLD. Obesity is a significant contributing factor to NAFLD progression^34,35^ therefore, it is likely that the protective effects are due to the secondary effects of obesity control. However, the lack of significant differences in fat accumulation and inflammation/fibrosis marker gene expression levels may be due to factors such as feeding periods and the timing of observations. Further detailed investigation is required to confirm the protective effects against NAFLD.

HFD-induced obesity in mice induces sarcopenia, which is associated with decreased muscle strength and mass. The grip strength test demonstrated that the typical decline in muscle strength was significantly mitigated by administration of heat-killed *F. fructosus* OS-1010. However, no structural differences in the amount of weight loss, or cross-sectional area per fiber were observed between the two groups. Non-contractile area such as IMAT is known to negatively impact muscle strength^25–27^. We examined the expression levels of the mature adipocyte markers, *Adipoq* and *Fabp4*, and found that these genes were significantly reduced in the HFD + OS-1010 group. From these results, it has been hypothesized that the attenuation of muscle weakness may be attributable to the suppression of IMAT accumulation. To further validate this hypothesis, direct quantification of adipocytes in muscle tissue would be necessary. Moreover, mitochondrial quality may contribute significantly to muscle function^36^. To gain insight, we analyzed the expression levels of genes related to mitochondria in the skeletal muscle and found a significant increase in the expression of the mitochondrial biosynthesis-related gene, *Sirt1*. Furthermore, there was an upward trend in CS activity, a reliable indicator of mitochondrial density. SIRT1 is a NAD^+^-dependent deacetylase that regulates mitochondrial biogenesis by deacetylating PGC-1α^30–32^. The significant increase in *Sirt1*, OXPHOS genes and the upward trend in CS activity suggest that mitochondrial biogenesis and/or turnover are enhanced in quadriceps. Although no significant change was observed in PGC-1α expression in present study, this suggests that the acetylation state may have changed. Therefore, it is required to measure the deacetylase activity of SIRT1, the concentration of NAD^+^, which is its activating substrate, as well as the acetylation of PGC-1α and the quantification of OXPHOS constituent proteins in the future. Furthermore, as mitochondrial biogenesis is also reportedly regulated by SIRT1-independent pathway (e.g. GCN5 acetyltransferase-mediated pathway^37^), a more comprehensive analysis is required. Similar research, that did not focus on SIRT1, has demonstrated that administration of heat-killed *Bifidobacterium breve* B-3 significantly increases the phosphorylated AMP-activated protein kinase, and mRNA expression of PGC-1α and cytochrome c oxidase genes in rat soleus, suggesting an effect on AMPK-PGC1α-mitochondrial biogenesis pathway^38^. In addition, heat-killed *B. breve* B-3 also induced an oxidative fiber type composition in rat gastrocnemius and increased grip strength. Although a direct comparison is difficult due to discrepancies in experimental designs (e.g. animal species and muscle tissue types), it is possible that heat-killed *F. fructosus* OS-1010 also increased mitochondrial energy productivity and changed in fiber type composition in skeletal muscle via promoting mitochondrial biogenesis. The investigation of the effect of heat-killed *F. fructosus* OS-1010 on skeletal muscle mitochondria in tissues with varying degrees of oxidative capacity, such as the soleus and gastrocnemius, could provide valuable insights into the true nature of its influence on skeletal muscle mitochondria.

The reason why administration of heat-killed *F. fructosus* OS-1010 caused an anti-obesity effect remains unclear in this study. Heat-killed *Lactiplantibacillus plantarum* L-137 decreases inflammation-related gene CD11c in the epididymal adipose tissue and exerts a transient anti-obesity effect in the early phase of obesity in mice on high-fat diet^6^. The transient anti-obesity effect was suggested to be exerted by temporarily suppressing the recruitment or differentiation of CD11c + M2 macrophages in adipose tissue and reducing production of growth/ differentiation factor 3, which inhibits lipolysis of adipose tissue and accelerates obesity^39^. Heat-killed *Lactiplantibacillus plantarum* K8, which reportedly has an anti-obesity effect on high-fat diet-induced obesity mice, has been suggested to control adipogenesis caused by FABP4 and lipogenic enzymes in gonadal adipose tissue via suppressing the expression of peroxisome proliferator-activated receptor γ and CCAAT/enhancer binding protein α^40^. Although the mechanisms underlying the anti-obesity effects suggested by the two studies mentioned above are completely different, molecular biological analysis of adipose tissue is a critical perspective for elucidating the mechanisms of anti-obesity effects. Therefore, further study focusing on adipose tissue is required to elucidate the mechanism of action of the anti-obesity effect of heat-killed *F. frucosus* OS-1010 in future. On the other hand, interestingly, live *Bifidobacterium breve* B-3, not heat-killed, has been suggested to exert anti-obesity effects via inducing mitochondrial biogenesis in skeletal muscle^38^. Mitochondria play a key role in energy metabolism, and numerous studies have explored their relationship with obesity^14,41–43^. Skeletal muscle, which has one of the highest energy consumptions in the body, is heavily dependent on mitochondria. The increased mitochondria may contribute to improving energy production efficiency in muscle cells and suppressing the accumulation of excess energy as fat. Furthermore, skeletal muscle is a significant tissue that consumes glucose in response to insulin. Given that SIRT1 is known to regulate insulin resistance in skeletal muscle^44–47^ the observed suppression of obesity may be partially related to increased energy consumption in skeletal muscle through the control of insulin resistance induced by increased SIRT1 expression. Moreover, FABP4, which was significantly downregulated in the HFD + OS-1010 group, has also been reported to improve insulin resistance by inhibiting its function^48^ and is associated with insulin resistance in skeletal muscle^49^. Directly evaluating mitochondrial function by measuring ATP levels in muscle tissue and quantifying blood insulin and glucose levels to evaluate insulin resistance may help to elucidate the mechanisms of the anti-obesity effect of heat-killed *F. fructosus* OS-1010.

The present study does not provide a comprehensive explanation for the observed upregulation of *Sirt1* in skeletal muscle. SIRT1 expression is known to be influenced by exercise^50^. It cannot be ruled out that differences in exercise levels between groups contributed to the observed changes in SIRT1 mRNA expression. Conversely, recent study has reported that when heat-killed *F. fructosus* OS-1010 is applied to human intestinal Caco-2 cells, and exosomes secreted by Caco-2 cells act on muscle cell C2C12, the number, area and membrane potential of mitochondria in C2C12 cells increases^13^. Caco-2-derived exosomes, which enhanced the number of mitochondria, contain miRNAs that alter the expression of SIRT1. While this result was based on an in vitro system using cells derived from humans, it cannot fully provide insight into the mechanism of the increase in SIRT1 expression observed in experiments using mice models. However, it demonstrates the potential of exosomes derived from the intestinal tract to act on distant muscle tissues and affect SIRT1 expression. It may be possible to clarify the contribution of exosomes by isolating and purifying them from the blood of mice that were administered *F. fructosus* OS-1010, and then evaluating their effects on C2C12 cultured cells.

In conclusion, this study demonstrated for the first time using a mouse model that the administration of heat-killed *F. fructosus* OS-1010 reduced diet-induced obesity. Furthermore, changes in several gene expressions and mitochondrial enzyme activity were observed, suggesting that the accumulation of IMAT in skeletal muscle and mitochondrial dysfunction are alleviated, and these findings may be associated with the suppression of muscle weakness induced by the administration of *F. fructosus* OS-1010. While the exact mechanism of its anti-obesity effects remains unclear, the enhanced mRNA expression levels of SIRT1 and the tendency toward increased mitochondrial density in quadriceps, as observed in this study, are consistent with previous in vitro study and may provide important clues for understanding the mechanism of anti-obesity effects.

## Methods

### Animal experiments

Wild-type male C57BL/6 mice (aged 8–12 weeks) were purchased from CLEA Japan, Inc. (Tokyo, Japan). The mice were housed under a 12-h light/dark cycle with stable temperature and humidity. After a three-week acclimatization period, the mice were randomly assigned to one of three groups (ND, HFD, or HFD containing heat-killed *F. fructosus* OS-1010), based on their body weight as an allocation factor. Each group comprised nine or twelve mice, housed three per cage. The experiment, from breeding to sacrifice, was performed twice, and the muscle (*n* = 8–9 animals/group) and liver (*n* = 9–12 animals/group) samples were collected in each round.

### Dietary intervention

The ND and HFD groups were fed a standard diet *ad libitum* (#D12450J, Research Diets, New Brunswick, NJ, USA) and HFD (60 kcal% fat, 7 kcal% sucrose; #D12492, Research Diets), respectively. The *F. fructosus* OS-1010 intake group (HFD + OS-1010) received HFD containing 2% heat-killed *F. fructosus* OS-1010 powder (customized #D12492, Research Diet). The calorie, carbohydrate, protein and fat composition of the diets given to the HFD + OS-1010 group is exactly the same as that of the diets given to the HFD group. To ensure optimal nutritional intake, the provided diets were replaced with fresh diets twice a week, and water was provided *ad libitum* which was changed once a week. Diet and water consumption were recorded at each replacement step.

### Grip strength test

Forelimb grip strength test was performed at the 12-week mark during the feeding period using a GPM-100B grip strength meter (MELQUEST, Toyama, Japan) by a blinded operator. Five measurements were taken per mouse, and the maximum value (in grams) was considered the grip strength.

### Micro-CT (µCT)

Liver fat percentage was assessed using a µCT scanner (Latheta LCT-200, Hitachi Aloka Medical, Tokyo, Japan). During scanning, the mice were anesthetized with 2–3% isoflurane. Fat accumulation was evaluated based on the contrast ratio between the spleen and visceral fat.

### Serum analysis

Serum AST, ALT, triglyceride, total cholesterol, LDL-C, and HDL-C levels were measured by Oriental Yeast Co., Ltd. (Tokyo, Japan). Serum samples indicating hemolysis were excluded from the analysis.

### Histological analysis

The quadriceps and liver were harvested. Immediately, the wet weights were weighed and fixed in 4% paraformaldehyde for 24 h. Paraffin-embedded tissue blocks were sectioned (3 μm) and stained with hematoxylin and eosin (HE). Digital images of full sections were captured using a BZ-X810 microscope (KEYENCE, Osaka, Japan). For quadriceps, the total cross-sectional area of the rectus femoris fibers was measured, and the number of fibers was counted from digital images using ImageJ software version 1.54d. For liver tissue, the total area of HE-negative oil droplets and the total liver section area were measured using ImageJ.

### Quantitative real-time PCR

Total RNA was extracted from tissue using TRIzol™ (Invitrogen, Waltham, MA, USA) and purified using NucleoSpin^®^ RNA Clean-Up (MACHEREY-NAGEL, Düren, Germany). cDNA was synthesized using a PrimeScript™ RT Reagent Kit with gDNA Eraser (Takara Bio, Shiga, Japan). Real time PCR were performed to determine the relative expression of the following genes: *p16*^*INK4a*^ (forward, 5ʹ-TGTTGAGGCTAGAGAGGATCTTG-3ʹ; reverse, 5ʹ-CGAATCTGCACCGTAGTTGAGC-3ʹ), *Col1a1* (forward, 5ʹ-CCTCAGGGTATTGCTGGACAAC-3ʹ; reverse, 5ʹ-CAGAAGGACCTTGTTTGCCAGG-3ʹ), *Col3a1* (forward, 5ʹ-GACCAAAAGGTGATGCTGGACAG-3ʹ; reverse, 5ʹ-CAAGACCTCGTGCTCCAGTTAG-3ʹ), *Cidea* (forward, 5ʹ-GGTGGACACAGAGGAGTTCTTTC-3ʹ; reverse, 5ʹ-CGAAGGTGACTCTGGCTATTCC-3ʹ), *Tnfα* (forward, 5ʹ-GGTGCCTATGTCTCAGCCTCTT-3ʹ; reverse, 5ʹ-GCCATAGAACTGATGAGAGGGA-3ʹ), *Adipoq* (forward, 5ʹ-GTGATGGCAGAGATGGCACT-3ʹ; reverse, 5ʹ-TCCTGTCTCACCCTTAGGACC-3ʹ), *Fabp4* (forward, 5ʹ-AAGGTGAAGAGCATCATAACCCT-3ʹ; reverse, 5ʹ-TCACGCCTTTCATAACACATTCC-3ʹ), *Sirt1* (forward, 5ʹ-GGAGCAGATTAGTAAGCGGCTTG-3ʹ; reverse, 5ʹ-GTTACTGCCACAGGAACTAGAGG − 3ʹ), *Pgc1α* (forward, 5ʹ-GAATCAAGCCACTACAGACACCG-3ʹ; reverse, 5ʹ-CATCCCTCTTGAGCCTTTCGTG-3ʹ), *Uqcrc1* (forward, 5ʹ-AGTGTGGATTGACGCTGGCAGT-3ʹ; reverse, 5ʹ-CCTCCTTCTCTAAGGCATTGCC-3ʹ), and *Atp5a1* (forward, 5ʹ-TGGTGAAGAGACTGACGGATGC-3ʹ; reverse, 5ʹ-TCAAAGCGTGCTTGCCGTTGTC-3ʹ). The expression levels of the target genes were normalized to 18S rRNA.

### CS activity assay

The prepared protein extraction solution was diluted with Tris-HCl buffer (pH 8.0), and 3 mM acetyl-CoA, 1 mM 5,5ʹ-dithiobis-(2-nitrobenzoate) (DTNB), and 1 mM oxaloacetic acid were added to make up the final concentrations. The reaction was performed at 25 °C for 1.5 min, and the initial reaction rate was calculated from the change in absorbance at 412 nm per minute. Results were normalized to total protein concentration and analyzed as relative activity.

### Statistical analysis

Statistical analyses were conducted using the Mann–Whitney *U* test for independent samples. Data are presented as means ± standard error of the mean, unless specified otherwise. Statistical significance was set at *P* < 0.05. All statistical analyses and graph generation were performed using GraphPad Prism version 10 (GraphPad Software, Inc., Boston, MA, USA).

## Data Availability

The datasets generated and analyzed in this study are available from the corresponding author upon reasonable request.

## References

[1] Ayivi, R. D. et al. Lactic acid bacteria: Food safety and human health applications. *Dairy* **1**, 202–232 (2020).

[2] Ma, L., Tu, H. & Chen, T. Postbiotics in human health: A narrative review. *Nutrients* **15**, 291 (2023). 36678162 10.3390/nu15020291 PMC9863882

[3] Vallejo-Cordoba, B., Castro-López, C., García, H. S., González-Córdova, A. F. & Hernández-Mendoza, A. Postbiotics and paraprobiotics: A review of current evidence and emerging trends. *Adv. Food Nutr. Res.* **94**, 1–34 (2020). 32892831 10.1016/bs.afnr.2020.06.001

[4] Ryu, S. et al. Postbiotic heat-killed lactobacilli modulates on body weight associated with gut microbiota in a pig model. *AMB Express*. **12**, 83 (2022). 35767074 10.1186/s13568-022-01424-8 PMC9243212

[5] Tanaka, Y. et al. Heat-killed *Lactiplantibacillus plantarum* Shinshu N-07 exerts antiobesity effects in Western diet-induced obese mice. *J. Appl. Microbiol.* **135**, lxae119 (2024). 38740521 10.1093/jambio/lxae119

[6] Yoshitake, R., Hirose, Y., Murosaki, S. & Matsuzaki, G. Heat-killed *Lactobacillus plantarum* L-137 attenuates obesity and associated metabolic abnormalities in C57BL/6 J mice on a high-fat diet. *Biosci. Microbiota Food Health*. **40**, 84–91 (2021). 33996364 10.12938/bmfh.2020-040 PMC8099634

[7] Hsieh, F. C. et al. Heat-killed and live: *Lactobacillus reuteri* GMNL-263 exhibit similar effects on improving metabolic functions in high-fat diet-induced obese rats. *Food Funct.* **7**, 2374–2388 (2016). 27163114 10.1039/c5fo01396h

[8] Cheng, Y. C. et al. Effects of heat-killed *Lactiplantibacillus plantarum* TWK10 on exercise performance, fatigue, and muscle growth in healthy male adults. *Physiol. Rep.* **11**, e15835 (2023). 37816697 10.14814/phy2.15835 PMC10564709

[9] Endo, A. et al. Fructophilic lactic acid bacteria, a unique group of fructose-fermenting microbes. *Appl. Environ. Microbiol.* **84**, e01290–e01218 (2018). 30054367 10.1128/AEM.01290-18 PMC6146980

[10] Alcántara-Hernández, R. J. et al. The bacterial community in ‘taberna’ a traditional beverage of Southern Mexico. *Lett. Appl. Microbiol.* **51**, 558–563 (2010). 21039665 10.1111/j.1472-765X.2010.02934.x

[11] Di Cagno, R., Filannino, P., Cantatore, V. & Gobbetti, M. Novel solid-state fermentation of bee-collected pollen emulating the natural fermentation process of bee bread. *Food Microbiol.* **82**, 218–230 (2019). 31027777 10.1016/j.fm.2019.02.007

[12] Takatani, N. & Endo, A. Viable fructophilic lactic acid bacteria present in honeybee-based food products. *FEMS Microbiol. Lett.* **368**, fnab150 (2021). 34850868 10.1093/femsle/fnab150

[13] Kashiwagi, R., Udono, M. & Katakura, Y. *Fructobacillus fructosus* OS-1010 strain stimulates intestinal cells to secrete exosomes that activate muscle cells. *Cytotechnology* **76**, 209–216 (2024). 38495295 10.1007/s10616-023-00610-1 PMC10940565

[14] Pileggi, C. A., Hooks, B. G., McPherson, R., Dent, R. R. M. & Harper, M. E. Targeting skeletal muscle mitochondrial health in obesity. *Clin. Sci.* **136**, 1081–1110 (2022). 10.1042/CS20210506 PMC9334731 35892309

[15] Li, J., Wu, H., Liu, Y. & Yang, L. High fat diet induced obesity model using four strains of mice: Kunming, C57bl/6, balb/c and Icr. *Exp. Anim.* **69**, 326–335 (2020). 32188837 10.1538/expanim.19-0148 PMC7445062

[16] Lei, F. et al. Evidence of anti-obesity effects of the pomegranate leaf extract in high-fat diet induced obese mice. *Int. J. Obes.* **31**, 1023–1029 (2007). 10.1038/sj.ijo.0803502 17299386

[17] Zhou, Q. et al. Beneficial effect of higher dietary fiber intake on plasma HDL-C and TC/HDL-C ratio among Chinese rural-to-urban migrant workers. *Int. J. Environ. Res. Public. Health*. **12**, 4726–4738 (2015). 25938914 10.3390/ijerph120504726 PMC4454936

[18] Zhang, X., Xu, G. B., Zhou, D. & Pan, Y. X. High-fat diet modifies expression of hepatic cellular senescence gene P16(INK4a) through chromatin modifications in adult male rats. *Genes Nutr.* **13**, 6 (2018). 29564021 10.1186/s12263-018-0595-5 PMC5853101

[19] Al-Qarni, R. et al. Validating candidate biomarkers for different stages of non-alcoholic fatty liver disease. *Medicine* **99**, e21463 (2020). 32898995 10.1097/MD.0000000000021463 PMC7478685

[20] Pellicano, A. J., Spahn, K., Zhou, P., Goldberg, I. D. & Narayan, P. Collagen characterization in a model of nonalcoholic steatohepatitis with fibrosis; a call for development of targeted therapeutics. *Molecules* **26**, 3316 (2021). 34205850 10.3390/molecules26113316 PMC8198364

[21] Sans, A. et al. The differential expression of Cide family members is associated with Nafld progression from steatosis to steatohepatitis. *Sci. Rep.* **9**, 7501 (2019). 31097771 10.1038/s41598-019-43928-7 PMC6522528

[22] Kakino, T. et al. Pivotal role of TNF-α in the development and progression of nonalcoholic fatty liver disease in a murine model. *Horm. Metab. Res.* **50**, 80–87 (2018). 28922680 10.1055/s-0043-118666

[23] Xie, W. et al. Mouse models of sarcopenia: Classification and evaluation. *J. Cachexia Sarcopenia Muscle*. **12**, 538–554 (2021). 33951340 10.1002/jcsm.12709 PMC8200444

[24] Jacobs, R. A., Díaz, V., Meinild, A. K., Gassmann, M. & Lundby, C. The C57Bl/6 mouse serves as a suitable model of human skeletal muscle mitochondrial function. *Exp. Physiol.* **98**, 908–921 (2013). 23180810 10.1113/expphysiol.2012.070037

[25] Therkelsen, K. E., Pedley, A., Hoffmann, U., Fox, C. S. & Murabito, J. M. Intramuscular fat and physical performance at the Framingham heart study. *Age (Omaha)*. **38**, 31 (2016). 10.1007/s11357-016-9893-2 PMC5005897 26899132

[26] Biltz, N. K. et al. Infiltration of intramuscular adipose tissue impairs skeletal muscle contraction. *J. Physiol.* **598**, 2669–2683 (2020). 32358797 10.1113/JP279595 PMC8767374

[27] Wang, L., Valencak, T. G. & Shan, T. Fat infiltration in skeletal muscle: Influential triggers and regulatory mechanism. *iScience* **27**, 109221 (2024). 38433917 10.1016/j.isci.2024.109221 PMC10907799

[28] Kopinke, D., Roberson, E. C. & Reiter, J. F. Ciliary Hedgehog signaling restricts injury-induced adipogenesis. *Cell* **170**, 340–351e12 (2017). 28709001 10.1016/j.cell.2017.06.035 PMC5617351

[29] Johnson, C. D., Zhou, L. Y. & Kopinke, D. A guide to examining intramuscular fat formation and its cellular origin in skeletal muscle. *JoVE* **183**, e63996 (2022). 10.3791/63996 PMC9741761 35695517

[30] Lagouge, M. et al. Resveratrol improves mitochondrial function and protects against metabolic disease by activating SIRT1 and PGC-1α. *Cell* **127**, 1109–1122 (2006). 17112576 10.1016/j.cell.2006.11.013

[31] Luen Tang, B. Sirt1 and the mitochondria. *Mol. Cells*. **39**, 87–95 (2016). 26831453 10.14348/molcells.2016.2318 PMC4757807

[32] Kong, S., Cai, B. & Nie, Q. PGC-1α affects skeletal muscle and adipose tissue development by regulating mitochondrial biogenesis. *Mol. Genet. Genom.* **297**, 621–633 (2022). 10.1007/s00438-022-01878-2 35290519

[33] Kundu, D. et al. p16 INK4A drives nonalcoholic fatty liver disease phenotypes in high fat diet fed mice through biliary E2F1/FOXO1/IGF-1 signaling. *Hepatology* **78**, 243–257 (2023). 36799449 10.1097/HEP.0000000000000307 PMC10410572

[34] Cho, E. J. et al. Body weight gain rather than body weight variability is associated with increased risk of nonalcoholic fatty liver disease. *Sci. Rep.* **11**, 14428 (2021). 34257374 10.1038/s41598-021-93883-5 PMC8277820

[35] Karjoo, S., Auriemma, A., Fraker, T. & Bays, H. E. Nonalcoholic fatty liver disease and obesity: An obesity medicine association (OMA) clinical practice statement (CPS) 2022. *Obes. Pillars*. **3**, 100027 (2022). 37990727 10.1016/j.obpill.2022.100027 PMC10661876

[36] Kerr, H. L. et al. Mouse sarcopenia model reveals sex- and age-specific differences in phenotypic and molecular characteristics. *J. Clin. Invest.* **134**, e172890 (2024). 39145448 10.1172/JCI172890 PMC11324300

[37] Philp, A. et al. Sirtuin 1 (SIRT1) deacetylase activity is not required for mitochondrial biogenesis or peroxisome proliferator-activated receptor-γ coactivator-1α (PGC-1α) deacetylation following endurance exercise. *J. Biol. Chem.* **286**, 30561–30570 (2011). 21757760 10.1074/jbc.M111.261685 PMC3162416

[38] Toda, K. et al. Heat-killed *Bifidobacterium breve* B-3 enhances muscle functions: Possible involvement of increases in muscle mass and mitochondrial biogenesis. *Nutrients* **12**, 219 (2020). 31952193 10.3390/nu12010219 PMC7019314

[39] Bu, Y. et al. Insulin regulates lipolysis and fat mass by upregulating growth/differentiation factor 3 in adipose tissue macrophages. *Diabetes* **67**, 1761–1772 (2018). 29945891 10.2337/db17-1201

[40] Jang, K. O. et al. Anti-obesity potential of heat-killed *Lactiplantibacillus plantarum* K8 in 3T3-L1 cells and high-fat diet mice. *Heliyon* **9**, e12926 (2023). 36699277 10.1016/j.heliyon.2023.e12926 PMC9868538

[41] Wang, Z., Yuan, D., Duan, Y., Li, S. & Hou, S. Key factors involved in obesity development. *Eat. Weight Disord.* **23**, 267–274 (2018). 28840575 10.1007/s40519-017-0428-3

[42] de Mello, A. H., Costa, A. B., Engel, J. D. G. & Rezin, G. T. Mitochondrial dysfunction in obesity. *Life Sci.* **192**, 26–32 (2018). 29155300 10.1016/j.lfs.2017.11.019

[43] Jun, L., Tao, Y. X., Geetha, T. & Babu, J. R. Mitochondrial adaptation in skeletal muscle: Impact of obesity, caloric restriction, and dietary compounds. *Curr. Nutr. Rep.* **13**, 500–515 (2024). 38976215 10.1007/s13668-024-00555-7 PMC11327216

[44] Schenk, S. et al. Sirt1 enhances skeletal muscle insulin sensitivity in mice during caloric restriction. *J. Clin. Invest.* **121**, 4281–4288 (2011). 21985785 10.1172/JCI58554 PMC3204844

[45] Liang, F. et al. Low-frequency electroacupuncture improves insulin sensitivity in obese diabetic mice through activation of SIRT1/PGC-1α in skeletal muscle. *Evid. Based Complement. Alternat. Med.* **2011**, 735297 (2011). 20981161 10.1155/2011/735297 PMC2964507

[46] Fröjdö, S. et al. Phosphoinositide 3-kinase as a novel functional target for the regulation of the insulin signaling pathway by SIRT1. *Mol. Cell. Endocrinol.* **335**, 166–176 (2011). 21241768 10.1016/j.mce.2011.01.008

[47] Zhang, H. H. et al. SIRT1 attenuates high glucose-induced insulin resistance via reducing mitochondrial dysfunction in skeletal muscle cells. *Exp. Biol. Med.* **240**, 557–565 (2015). 10.1177/1535370214557218 PMC4935266 25710929

[48] Furuhashi, M. et al. Treatment of diabetes and atherosclerosis by inhibiting fatty-acid-binding protein aP2. *Nature* **447**, 959–965 (2007). 17554340 10.1038/nature05844 PMC4076119

[49] Okura, T. et al. Postprandial fatty acid-binding protein 4 is associated with muscle insulin resistance. *Diabetologia* **67**, 2304–2315 (2024). 39060707 10.1007/s00125-024-06222-4

[50] Vargas-Ortiz, K., Pérez-Vázquez, V. & Macías-Cervantes, M. H. Exercise and sirtuins: A way to mitochondrial health in skeletal muscle. *Int. J. Mol. Sci.* **20**, 2717 (2019). 31163574 10.3390/ijms20112717 PMC6600260

